# Epidemiologic and Clinical Characteristics of Monkeypox Cases — United States, May 17–July 22, 2022

**DOI:** 10.15585/mmwr.mm7132e3

**Published:** 2022-08-12

**Authors:** David Philpott, Christine M. Hughes, Karen A. Alroy, Janna L. Kerins, Jessica Pavlick, Lenore Asbel, Addie Crawley, Alexandra P. Newman, Hillary Spencer, Amanda Feldpausch, Kelly Cogswell, Kenneth R. Davis, Jinlene Chen, Tiffany Henderson, Katherine Murphy, Meghan Barnes, Brandi Hopkins, Mary-Margaret A. Fill, Anil T. Mangla, Dana Perella, Arti Barnes, Scott Hughes, Jayne Griffith, Abby L. Berns, Lauren Milroy, Haley Blake, Maria M. Sievers, Melissa Marzan-Rodriguez, Marco Tori, Stephanie R. Black, Erik Kopping, Irene Ruberto, Angela Maxted, Anuj Sharma, Kara Tarter, Sydney A. Jones, Brooklyn White, Ryan Chatelain, Mia Russo, Sarah Gillani, Ethan Bornstein, Stephen L. White, Shannon A. Johnson, Emma Ortega, Lori Saathoff-Huber, Anam Syed, Aprielle Wills, Bridget J. Anderson, Alexandra M. Oster, Athalia Christie, Jennifer McQuiston, Andrea M. McCollum, Agam K. Rao, María E. Negrón, CDC Multinational Monkeypox Response Team, CDC Multinational Monkeypox Response Team, Isabel Griffin, Mohammed Khan, Yasmin Ogale, Emily Sims, R. Ryan Lash, Jeanette J. Rainey, Kelly Charniga, Michelle A. Waltenburg, Patrick Dawson, Laura A.S. Quilter, Julie Rushmore, Mark R. Stenger, Rachel E. Kachur, Florence Whitehill, Kelly A. Jackson, Jim Collins, Kimberly Signs, Gillian Richardson, Julie Hand, Emily Spence-Davizon, Brandi Steidley, Matthew Osborne, Susan Soliva, Sabrina Cook, Leslie Ayuk-Takor, Christina Willut, Alexandria Snively, Nicholas Lehnertz, Daniela N. Quilliam, Miranda Durham, Iris R. Cardona-Gerena, Linda J. Bell, Environmental Control, Marina Kuljanin, Suzanne Gibbons-Burgener, Ryan Westergaard, Lynn E. Sosa, Monica Beddo, Matthew Donahue, Samir Koirala, Courtney Dewart, Jade Murray-Thompson, Lilian Peake, Michelle L. Holshue, Atul Kothari, Jamie Ahlers, Lauren Usagawa, Megan Cahill, Erin Ricketts, Mike Mannell, Farah S. Ahmed, Bethany Hodge, Brenton Nesemeier, Katherine Guinther, Madhu Anand, Jennifer L. White, Joel A. Ackelsberg, Ellen H. Lee, Devin Raman, Carmen Brown, Nicole Burton, Sarakay Johnson

**Affiliations:** ^1^Epidemic Intelligence Service; ^2^CDC Monkeypox Response; ^3^New York City Department of Health and Mental Hygiene, New York, New York; ^4^Chicago Department of Health, Chicago, Illinois; ^5^Georgia Department of Health; ^6^Philadelphia Department of Public Health, Philadelphia, Pennsylvania; ^7^New York State Department of Health; ^8^Oregon Department of Health; ^9^Texas Department of State Health Services; ^10^Maryland Department of Health; ^11^Michigan Department of Health and Human Services; ^12^Louisiana Department of Health; ^13^Colorado Department of Public Health and Environment; ^14^Massachusetts Department of Public Health; ^15^Tennessee Department of Health; ^16^DC Department of Health; ^17^Illinois Department of Public Health; ^18^Minnesota Department of Health; ^19^Rhode Island Department of Health; ^20^Indiana State Department of Health; ^21^Southern Nevada Health District, Las Vegas, Nevada; ^22^New Mexico Department of Health; ^23^Puerto Rico Department of Health; ^24^South Carolina Department of Health and Environmental Control; ^25^Laboratory Leadership Service, CDC; ^26^Arizona Department of Health Services; ^27^Wisconsin Department of Health Services; ^28^Ohio Department of Health; ^29^Connecticut Department of Public Health; ^30^Career Epidemiology Field Officer Training Program, CDC ^31^Missouri Department of Health and Senior Services; ^31^Salt Lake County Health Department, Salt Lake City, Utah; ^32^Pennsylvania Department of Health.; CDC; CDC; CDC; CDC; CDC; CDC; CDC; CDC; CDC; CDC; CDC; CDC; CDC; CDC; CDC; Michigan Department of Health and Human Services; Michigan Department of Health and Human Services; Louisiana Department of Health; Louisiana Department of Health; Colorado Department of Public Health and Environment; Colorado Department of Public Health and Environment; Massachusetts Department of Public Health; Massachusetts Department of Public Health; Joanna Shaw-KaiKai; Nashville Metro Public Health Department; Nashville Metro Public Health Department; DC Department of Health; DC Department of Health; Indiana Department of Health; Minnesota Department of Health; Rhode Island Department of Health; New Mexico Department of Health; Puerto Rico Department of Health; South Carolina Department of Health; Maricopa County Department of Health; Wisconsin Department of Health Services; Wisconsin Department of Health Services; Connecticut Department of Public Health; Missouri Department of Health and Senior Services; Nebraska Department of Health and Human Services; Nebraska Department of Health and Human Services; Ohio Department of Health, Career Epidemiology Field Officer, CDC; Utah Department of Health and Human Services; Virginia Department of Health; Washington Department of Health; Arkansas Department of Health; Delaware Department of Health and Social Services; Hawaii Department of Health; Idaho Division of Public Health; North Carolina Department of Health and Human Services; Oklahoma State Department of Health; Kansas Department of Health and Environment; Kentucky Department for Public Health; North Dakota Department of Health; West Virginia Bureau for Public Health; New York State Department of Health; New York State Department of Health; New York City Department of Health and Mental Hygiene; New York City Department of Health and Mental Hygiene; Southern Nevada Health District; Pennsylvania Department of Health; New York City Department of Health and Mental Hygiene; Metro Public Health Department–Nashville.

Monkeypox, a zoonotic infection caused by an orthopoxvirus, is endemic in parts of Africa. On August 4, 2022, the U.S. Department of Health and Human Services declared the U.S. monkeypox outbreak, which began on May 17, to be a public health emergency (*1**,**2*). After detection of the first U.S. monkeypox case), CDC and health departments implemented enhanced monkeypox case detection and reporting. Among 2,891 cases reported in the United States through July 22 by 43 states, Puerto Rico, and the District of Columbia (DC), CDC received case report forms for 1,195 (41%) cases by July 27. Among these, 99% of cases were among men; among men with available information, 94% reported male-to-male sexual or close intimate contact during the 3 weeks before symptom onset. Among the 88% of cases with available data, 41% were among non-Hispanic White (White) persons, 28% among Hispanic or Latino (Hispanic) persons, and 26% among non-Hispanic Black or African American (Black) persons. Forty-two percent of persons with monkeypox with available data did not report the typical prodrome as their first symptom, and 46% reported one or more genital lesions during their illness; 41% had HIV infection. Data suggest that widespread community transmission of monkeypox has disproportionately affected gay, bisexual, and other men who have sex with men and racial and ethnic minority groups. Compared with historical reports of monkeypox in areas with endemic disease, currently reported outbreak-associated cases are less likely to have a prodrome and more likely to have genital involvement. CDC and other federal, state, and local agencies have implemented response efforts to expand testing, treatment, and vaccination. Public health efforts should prioritize gay, bisexual, and other men who have sex with men, who are currently disproportionately affected, for prevention and testing, while addressing equity, minimizing stigma, and maintaining vigilance for transmission in other populations. Clinicians should test patients with rash consistent with monkeypox,^†^ regardless of whether the rash is disseminated or was preceded by prodrome. Likewise, although most cases to date have occurred among gay, bisexual, and other men who have sex with men, any patient with rash consistent with monkeypox should be considered for testing. CDC is continually evaluating new evidence and tailoring response strategies as information on changing case demographics, clinical characteristics, transmission, and vaccine effectiveness become available.^§^

On June 3, 2022, CDC released a case report form for health departments to report monkeypox cases. Data collected include possible exposures during the 3 weeks preceding symptom onset, symptoms during the illness course, and distribution of rash, defined as at least one lesion on the skin or mucous membranes. To describe epidemiologic and clinical characteristics, CDC analyzed case report form data for probable or confirmed cases^¶^ initially reported through July 22, 2022; to allow for reporting delay, data received through July 27 were included. Analyses were restricted to cases for which relevant data were available. This activity was reviewed by CDC and was conducted consistent with applicable federal law and CDC policy.**

During May 17–July 22, 2022, a total of 2,891 U.S. monkeypox cases were reported by 43 states, Puerto Rico, and DC; the number of reported cases increased rapidly during this time (Figure). Case report forms including, at minimum, age and gender identity were received for 1,195 (41%) cases; these cases are described in this report. Median age was 35 years (IQR = 30–41 years). Nearly all (99%) persons with case report forms available were men (cisgender and transgender) (Table 1). Among 1,054 cases for which race and ethnicity were reported, 41% occurred among White persons, 28% among Hispanic persons, and 26% among Black persons. Based on information available in case report forms, the percentage of cases among Black persons increased from 12% (29 of 248) during May 17–July 2 to 31% (247 of 806) during July 3–22, and the percentage among Hispanic persons decreased from 33% (82 of 248) to 27% (214 of 806) and among White persons from 49% (121 of 248) to 38% (307 of 806).

**FIGURE:**
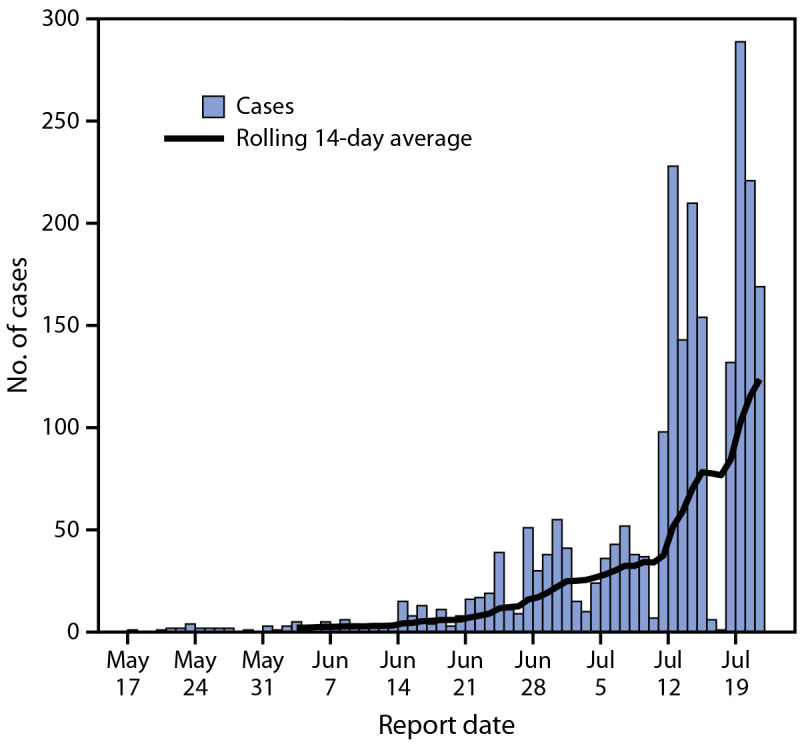
Monkeypox cases, by report date* — United States, May 17–July 22, 2022 * Includes either the positive laboratory test report date, CDC call center reporting date, or date of case data entry into CDC’s emergency response common operating platform.

**TABLE 1:** Characteristics of persons with monkeypox — United States, May 17–July 22, 2022

Characteristic (no. with available information)	No. (%)*
**Total**	**1,195 (100)**
**Gender identity (1,195)**	
Man	1,178 (98.7)
Transgender man	3 (0.3)
Woman	5 (0.4)
Transgender woman	5 (0.4)
Prefer not to answer	4 (0.3)
Missing	0 (—)
**Race and ethnicity (1,054)**	
Asian, non-Hispanic	48 (4.6)
Black, non-Hispanic	276 (26.2)
White, non-Hispanic	428 (40.6)
Hispanic	296 (28.1)
Multiple races, non-Hispanic	6 (0.6)
Missing	141

* Percentages calculated using nonmissing data.

Among 241 cases (20%) with reported classification by health departments as being travel-associated or locally acquired, 178 (74%) were classified as locally acquired. The percentage of locally acquired cases increased from 51% (33 of 65) during May 17–July 2 to 82% (145 of 175) during July 3–22.

Among 358 (30%) men (cisgender and transgender) with information on recent sexual behaviors and gender of sex partners available, 337 (94%) reported sex or close intimate contact with a man during the 3 weeks before symptom onset; 16 (4%) reported no such contact. Among 291 men who reported information about their male sexual partners during the 3 weeks preceding symptom onset, 80 (27%) reported one partner, 113 (40%) reported two to four partners, 42 (14%) reported five to nine partners, and 56 (19%) reported 10 or more partners. Among 86 men with information reported, 33 (38%) reported group sex, defined as sex with more than two persons, at a festival, group sex event, or sex party.

The most frequently reported signs and symptoms included rash (100%), fever (63%), chills (59%), and lymphadenopathy (59%) (Table 2). Reported rectal symptoms included purulent or bloody stools (21%), rectal pain (22%), and rectal bleeding (10%). Among 291 persons with available information about their first symptoms, 58% reported at least one prodromal symptom^††^; for the 42% of patients without prodromal symptoms, illness began with a rash.

**TABLE 2:** Symptoms and rash among persons with monkeypox — United States, May 17–July 22, 2022

Characteristic	Ever experienced during illness* (N = 1,007)			Initially experienced^†^ (N = 461)		
	No. (%)^§^		No. missing	No. (%)^§^		No. missing
	Yes	No		Yes	No	
**Symptoms**						
Rash^¶^	1,004 (100.0)	0 (—)	3	121 (41.6)	170 (58.4)	170
Fever	596 (63.3)	345 (36.7)	66	120 (41.2)	171 (58.8)	170
Chills	550 (59.1)	381 (40.9)	76	48 (16.5)	243 (83.5)	170
Lymphadenopathy	545 (58.5)	387 (41.5)	75	23 (7.9)	268 (92.1)	170
Malaise	531 (57.1)	399 (42.9)	77	24 (8.2)	267 (91.8)	170
Myalgia	507 (55)	415 (45)	85	13 (4.5)	278 (95.5)	170
Headache	469 (50.8)	454 (49.2)	84	27 (9.3)	264 (90.7)	170
Rectal pain	201 (21.9)	715 (78.1)	91	0 (—)	291 (100.0)	170
Pus or blood in stools	184 (20.5)	713 (79.5)	110	0 (—)	291 (100.0)	170
Abdominal pain	96 (11.5)	742 (88.5)	169	1 (0.3)	290 (99.7)	170
Rectal bleeding	90 (10.0)	810 (90.0)	107	0 (—)	291 (100.0)	170
Tenesmus	90 (10.0)	809 (90.0)	108	2 (0.7)	289 (99.3)	170
Vomiting or nausea	83 (9.2)	817 (90.8)	107	0 (—)	291 (100.0)	170
**Rash sites**						
Genitals	333 (46.4)	385 (53.6)	289	214 (55.7)	170 (44.3)	77
Arms	284 (39.6)	434 (60.4)	289	20 (5.2)	364 (94.8)	77
Face	276 (38.4)	442 (61.6)	289	94 (24.5)	290 (75.5)	77
Legs	265 (36.9)	453 (63.1)	289	18 (4.7)	366 (95.3)	77
Perianal	225 (31.3)	493 (68.7)	289	86 (22.4)	298 (77.6)	77
Mouth, lips, or oral mucosa	179 (24.9)	539 (75.1)	289	99 (25.8)	285 (74.2)	77
Palms of hands	157 (21.9)	561 (78.1)	289	13 (3.4)	371 (96.6)	77
Trunk	156 (21.7)	562 (78.3)	289	14 (3.6)	370 (96.4)	77
Neck	130 (18.1)	588 (81.9)	289	33 (8.6)	351 (91.4)	77
Head	97 (13.5)	621 (86.5)	289	8 (2.1)	376 (97.9)	77
Soles of feet	77 (10.7)	641 (89.3)	289	1 (0.3)	383 (99.7)	77

* Symptoms experienced up until the time of interview.

^†^ Symptoms reported by persons with monkeypox as their first symptoms during their illness or the body location where rash first appeared.

^§^ Percentages calculated using nonmissing data.

^¶^ Rash includes at least one lesion affecting the skin or mucous membranes.

Rash was most frequently reported on the genitals (46%), arms (40%), face (38%), and legs (37%); among 718 persons with monkeypox who reported body regions with rash, 238 (33%) reported rash in one region, 126 (18%) in two regions, 98 (14%) in three regions, and 256 (36%) in four or more regions. Among 104 persons with information on the number of lesions, 88% of cases involved fewer than 50 lesions.

Among 334 persons with data available on HIV status, 136 (41%) had HIV infection. Among 954 persons with hospitalization data available, 77 (8%) patients were hospitalized because of their illness. No deaths were reported. Among 339 persons with vaccination status available, 48 (14%) reported previous receipt of smallpox vaccine, including 11 (23%) who received 1 of 2 JYNNEOS doses during the current outbreak, 11 (23%) who received pre-exposure prophylaxis at an unknown time before the current outbreak, and 26 (54%) who did not provide information about when vaccine was administered. Among the recently vaccinated persons with monkeypox, at least one experienced symptoms >3 weeks after their first JYNNEOS dose.

## Discussion

Current findings indicate that community transmission of monkeypox is widespread and is disproportionately affecting gay, bisexual, and other men who have sex with men; this is consistent with data reported from other countries (*3*). Public health efforts to slow monkeypox transmission among gay, bisexual, and other men who have sex with men require addressing challenges that include homophobia, stigma, and discrimination. Although the largest proportion of cases have occurred in White persons, Black and Hispanic persons, who represent approximately one third (34%) of the general population (*4*), accounted for more than one half (54%) of monkeypox cases in persons for whom information on race and ethnicity is available; further, the proportion of cases among Black persons has increased during recent weeks. Ensuring equity in approaches to monkeypox testing, treatment, and prevention is critical, and taking actions to minimize stigma related to monkeypox can reduce barriers to seeking care and prevention. The data presented in this report provide insights into early transmission; however, ongoing surveillance is essential to monitor future transmission trends and assess the impacts among different communities.

These data can guide clinical considerations for evaluating persons for monkeypox. Typically, monkeypox begins with a febrile prodrome, which might include malaise, chills, headache, or lymphadenopathy, followed by a disseminated rash that often includes the palms and soles (*5*). Although most cases in this report included these features, 42% of persons did not report prodromal symptoms, and 37% did not report fever by the time of interview. Genital rash, although reported in fewer than one half of cases, was common; 36% of persons developed rash in four or more body regions. Other recent reports describe similar clinical characteristics (*6*,*7*). Clinicians should be vigilant for patients with rash consistent with monkeypox, regardless of whether the rash is disseminated or was preceded by prodrome. Likewise, although most cases to date have occurred among gay, bisexual, and other men who have sex with men, any patient, regardless of sexual or gender identity, with rash consistent with monkeypox should be considered for testing because close physical contact with an infectious person or exposure to contaminated materials such as clothing or bedding can result in transmission.

A substantial proportion of monkeypox cases have been reported among persons with HIV infection, and efforts are underway to characterize monkeypox clinical outcomes among these persons. Recent reports have found that concurrent sexually transmitted infections were common in persons with monkeypox (*3*,*7*). Clinicians and health officials implementing monkeypox education, testing, and prevention efforts should also incorporate recommended interventions for other conditions occurring among gay and bisexual men, including HIV infection, sexually transmitted infections, substance use, and viral hepatitis^§§ ^(*8*). 

On May 23, 2022, CDC launched an emergency response for monkeypox. This response includes educating providers and the public, expanding laboratory testing, outlining prevention strategies, and promoting the use of medical countermeasures for treatment and postexposure prophylaxis. CDC is supporting state, tribal, local, and territorial health departments through guidance and technical assistance. Testing capacity was rapidly expanded through CDC’s Laboratory Response Network and commercial laboratories, with national capacity estimates of 80,000 tests per week by July 18.^¶¶^

Because of long-standing investments in medical countermeasures for potential smallpox events, licensed vaccines and therapeutics for monkeypox are held in the U.S. Department of Health and Human Services Strategic National Stockpile. A national vaccine strategy was developed to equitably expand vaccination in areas experiencing high numbers of monkeypox cases and contacts. Two vaccines are available in the United States.*** As of August 3, more than 1 million doses of JYNNEOS, a nonreplicating, live virus vaccine (https://www.fda.gov/media/131078/download) had been allocated to jurisdictions, and approximately 14,700 courses of oral tecovirimat (TPOXX) had been distributed to jurisdictions and providers. 

The findings in this report are subject to at least three limitations. First, this analysis includes only 41% of U.S. monkeypox cases reported through July 22 and might not be representative of all cases. Jurisdictions with high numbers of cases without submitted case report forms were more racially and ethnically diverse according to U.S. Census Bureau data; therefore, persons from racial and ethnic minority groups might be more disproportionately affected than indicated by these data. Second, even on submitted case report forms, data for variables such as timing of vaccination, sexual behaviors, HIV status, reason for hospitalization, and whether cases were travel-associated were frequently missing; data might also not reflect symptoms or outcomes occurring after the interview. Finally, persons with monkeypox who have mild symptoms might be less likely to seek care or initiate testing and could be underrepresented in this analysis.

CDC is continually evaluating new evidence and tailoring response strategies as information on changing case demographics, clinical characteristics, transmission, and vaccine effectiveness become available. Public health efforts should prioritize gay, bisexual, and other men who have sex with men, who are currently disproportionately affected for prevention and testing, address equity, and minimize stigma, while maintaining vigilance for transmission in other populations. Clinicians should test persons with rash consistent with monkeypox, regardless of whether the rash is disseminated or was preceded by prodrome.

**Summary**

**What is already known about this topic?**

A global monkeypox outbreak began in 2022.

**What is added by this report?**

Among U.S. monkeypox cases with available data, 99% occurred in men, 94% of whom reported recent male-to-male sexual or close intimate contact; racial and ethnic minority groups appear to be disproportionately affected. Clinical presentations differed from typical monkeypox, with fewer persons experiencing prodrome and more experiencing genital rashes.

**What are the implications for public health practice?**

Public health efforts should prioritize gay, bisexual, and other men who have sex with men, who are currently disproportionately affected, for prevention and testing, address equity, and minimize stigma, while maintaining vigilance for transmission in other populations. Clinicians should test persons with rash consistent with monkeypox, regardless of whether the rash is disseminated or was preceded by prodrome

## Footnotes

* These authors contributed equally to this report.

†
https://www.cdc.gov/poxvirus/monkeypox/symptoms.html

§
https://www.cdc.gov/poxvirus/monkeypox/index.html

¶ A probable case was defined as illness for which there was no suspicion of other recent orthopoxvirus exposure and one of the following: 1) detection of orthopoxvirus DNA by polymerase chain reaction testing of a clinical specimen, 2) evidence of orthopoxvirus antigen using immunohistochemical staining or visualization by electron microscopy, or 3) demonstration of detectable levels of antiorthopoxvirus immunoglobulin M antibody during the 4–56 days after rash onset. A confirmed case was defined as 1) the presence of *Monkeypox virus* DNA by polymerase chain reaction testing or Next-Generation sequencing of a clinical specimen or 2) isolation of *Monkeypox virus* in culture from a clinical specimen.

** 45 C.F.R. part 46, 21 C.F.R. part 56; 42 U.S.C. Sect. 241(d); 5 U.S.C. Sect. 552a; 44 U.S.C. Sect. 3501 et seq.

†† Prodrome defined as at least one of the following: fever, myalgias, malaise, headaches, lymphadenopathy, or chills occurring as first symptom, not accompanied by a rash.

§§
https://www.cdc.gov/msmhealth/index.htm

¶¶
https://www.hhs.gov/about/news/2022/06/22/hhs-expanding-monkeypox-testing-capacity-five-commercial-laboratory-companies.html

***
https://www.cdc.gov/poxvirus/monkeypox/considerations-for-monkeypox-vaccination.html
